# Response: Commentary: Commonly Used Anesthesia/Euthanasia Methods for Brain Collection Differentially Impact MAPK Activity in Male and Female C57BL/6 Mice

**DOI:** 10.3389/fncel.2019.00379

**Published:** 2019-08-14

**Authors:** Mee Jung Ko, Richard M. van Rijn

**Affiliations:** ^1^Department of Medicinal Chemistry and Molecular Pharmacology, College of Pharmacy, West Lafayette, IN, United States; ^2^Purdue Interdisciplinary Life Sciences Graduate Program, West Lafayette, IN, United States; ^3^Purdue Institute for Integrative Neuroscience, West Lafayette, IN, United States

**Keywords:** ERK, JNK, p38, ketamine, isoflurane, carbon dioxide, central nervous system

We are responding to commentary by Kohtala and Rantamaki (2019) on our paper “Commonly Used Anesthesia/Euthanasia Methods for Brain Collection Differentially Impact MAPK Activity in Male and Female C57BL/6 Mice” (Ko et al., 2019). We welcome the expansion of the role of euthanasia methods on MAPK activation the authors provide in their commentary, and we apologize for our oversight in not discussing their recent relevant work in our paper. We are pleased that our open-access paper is being noticed by the scientific community and that the authors were able to rapidly comment on the paper. The general point of the authors is that our findings and interpretation do not align with their observations. For example, whereas we observed relatively strong ERK activity following a short (5 min) induction of anesthesia with isoflurane (Ko et al., 2019), Kohtala et al. observed a decrease in ERK activity when mice were exposed to isoflurane for 30 min relative to their “sham” mice (Kohtala et al., 2016). Overall, we echo the author's discussion that time, method and temperature all impact MAPK activity, and that changes in MAPK activity have a temporal component to them.

There are however several points we want to specifically address. Firstly, the authors propose that we did not properly design our study to compare the different euthanasia methods stating “the authors did not consider the effects of injection stress, since the decapitation, CO_2_, and isoflurane groups were not treated with a saline injection.” The objective of our study was to compare how common methods of anesthesia/euthanasia currently being used impact MAPK activity. Thus, logically we did not inject CO_2_ and isoflurane groups with saline as that is not an accurate reproduction of how one would carry out such an experiment in the field. Similarly, our ketamine/xylazine treatment time was chosen to be around the time it would take to properly sedate a mouse and to initiate and finish a transcardial perfusion. The use of heating pads during transcardial perfusion is not that common and thus was not included in our experimental design. Certainly, future experimenters can compare all methods at the exact same 5–10 min interval and may find different trends than what we found using our chosen design.

Secondly, the authors question our methodology and make the false assumption that our larger group size were obtained by “collecting bilateral samples from the same animals.” As described in our method section to test the reproducibility of our study we performed a separate study (i.e., separate animals at a different time of the year), which resulted in a similar trend in ERK activation for the tested methods (we refer the reader to Supplementary figure S1 in the original paper). This brings us to a third concern by the authors that our method section did not clearly describe our use of phosphatase inhibitors. To clarify, we used the HALT protease and phosphatase inhibitor from Thermo Scientific, purchased from Fisher Scientific with the catalog number #1861280, which is listed in the paper, but was inadequately described in the text as a protease inhibitor. We like to stress that the corresponding author information provided within our paper is correct and we would be happy to provide additional details on our methodology or clarify other concerns. Overall, we firmly stand behind the rigor of our experimental design and validity of our results. However, we surely do not claim that our findings are the definitive answer to the question of how anesthesia/euthanasia affects MAPK activity.

The authors end their commentary with the recommendation that “decapitation, or perhaps microwave irradiation remains the most optimal choice for animal experiments where protein phosphorylation is investigated.” We would provide feedback to this recommendation that most Institutional Animal Care and Use Committees (IACUCs) would not readily support the use of unanesthetized decapitation. In our study, isoflurane anesthesia followed by decapitation produced statistically identical results to unanesthetized decapitation. If, as argued by the authors, the MAPK activity level we observed for decapitation most closely resembles normal baseline MAPK activity, then, based on our observations, an IACUC would most likely recommend the use of isoflurane prior to decapitation to minimize pain to the animal.

## Author Contributions

All authors listed have made a substantial, direct and intellectual contribution to the work, and approved it for publication.

### Conflict of Interest Statement

The authors declare that the research was conducted in the absence of any commercial or financial relationships that could be construed as a potential conflict of interest.
